# The regulation and biosynthesis of antimycins

**DOI:** 10.3762/bjoc.9.290

**Published:** 2013-11-19

**Authors:** Ryan F Seipke, Matthew I Hutchings

**Affiliations:** 1 School of Biological Sciences, University of East Anglia, Norwich Research Park, Norwich, NR4 7TJ, United Kingdom; 2 School of Molecular and Cellular Biology, Garstang Buildling, Faculty of Biological Sciences, University of Leeds, Leeds, LS2 9JT, United Kingdom

**Keywords:** antimycins, gene regulation, genome mining, natural products, Streptomyces

## Abstract

Antimycins (>40 members) were discovered nearly 65 years ago but the discovery of the gene cluster encoding antimycin biosynthesis in 2011 has facilitated rapid progress in understanding the unusual biosynthetic pathway. Antimycin A is widely used as a piscicide in the catfish farming industry and also has potent killing activity against insects, nematodes and fungi. The mode of action of antimycins is to inhibit cytochrome c reductase in the electron transport chain and halt respiration. However, more recently, antimycin A has attracted attention as a potent and selective inhibitor of the mitochondrial anti-apoptotic proteins Bcl-2 and Bcl-x_L_. Remarkably, this inhibition is independent of the main mode of action of antimycins such that an artificial derivative named 2-methoxyantimycin A inhibits Bcl-x_L_ but does not inhibit respiration. The Bcl-2/Bcl-x_L_ family of proteins are over-produced in cancer cells that are resistant to apoptosis-inducing chemotherapy agents, so antimycins have great potential as anticancer drugs used in combination with existing chemotherapeutics. Here we review what is known about antimycins, the regulation of the *ant* gene cluster and the unusual biosynthetic pathway.

## Review

It is estimated that around 60% of all known antibiotics are derived from secondary metabolites made by filamentous actinomycete bacteria, most notably *Streptomyces* species [1]. *Streptomyces* species are predominantly known as saprophytic soil bacteria that have a complex differentiating life cycle. The life cycle begins with spore germination and outgrowth of a substrate mycelium and ends with the production of reproductive aerial hyphae, which undergo cell division to form chains of unigenomic spores [2]. Aerial hyphae production and sporulation is triggered by nutritional stress and is accompanied by the production of secondary metabolites. These specialised metabolites likely function both as chemical weapons against competing organisms in the soil and as signaling molecules to neighbouring microbes [3]. In recent years genome sequencing has revealed that each *Streptomyces* species encodes many more specialised metabolites than it makes in laboratory culture, leading to new efforts to activate these so-called “silent pathways.” The number of known antibiotics made by *Streptomyces* species is likely to grow rapidly with the advent of genome mining approaches, which start by identifying promising specialised metabolite gene clusters in whole genome sequences and then inducing their expression through chemical or genetic manipulation of the gene cluster in the native or a heterologous host. This approach has already been used to identify novel chemical scaffolds of antibiotics produced by well-studied *Streptomyces* species [4–6] and to identify the biosynthetic gene clusters for commercially important antibiotics [7–11]. The latter allows cloning, optimisation and engineering of such pathways to generate new derivatives with improved pharmacological properties.

**Figure 1:**
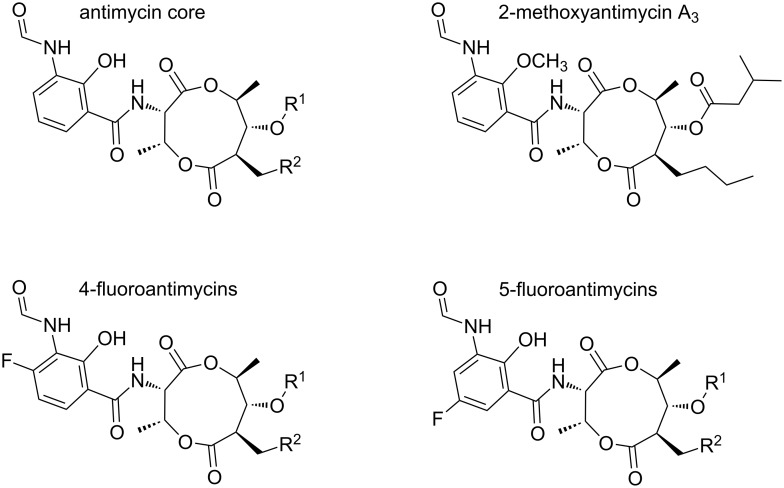
Antimycins: Antimycins A_1_, A_2_, A_3_, and A_4_ and non-natural antimycins referenced in the text. Antimycin A_1_, R^1^ = COCH(CH_3_)CH_2_CH_3_, R^2^ = (CH_2_)_4_CH_3_; Antimycin A_2_, R^1^ = COCH(CH_3_)_2_, R^2^ = (CH_2_)_4_CH_3_; Antimycin A_3_, R^1^ = COCH_2_CH(CH_3_)_2_, R^2^ = (CH_2_)_2_CH_3_; Antimycin A_4_, R^1^ = COCH(CH_3_)_2_, R^2^ = (CH_2_)_2_CH_3_. For a recent summary of the chemical diversity in the antimycin family see [25].

We recently sequenced the genome of *Streptomyces albus* S4, which we isolated from the cuticle of the leaf-cutter ant *Acromyrmex octospinosus* [12–13]. Using genome-mining strategies, we identified the biosynthetic gene cluster for a group of compounds called antimycins that were discovered more than 60 years ago [7,14]. Antimycins, including structurally related uranchimycins, kitamycins and splenocins have unique structures comprising a nine-membered dilactone core conjugated to a rare 3-formamidosalicylic acid moiety and they comprise more than 40 known members (Figure 1) [15–22]. Antimycins can undergo base-catalysed decomposition resulting in the production of volatile blastmycinones and butenolides [23]. The main mode of action of antimycins is to inhibit cytochrome c reductase, an enzyme in the electron transport chain in mitochondria and bacteria and as such they are bioactive against a wide range of oragnisms including fish, fungi, insects and nematodes [24].

Antimycins are widely used as a piscicide (brandname Fintrol) in the catfish farming industry. Catfish are easy to farm and they provide an inexpensive source of food in large parts of Asia and in the Southern USA. Catfish are relatively insensitive to antimycins and Fintrol is used to selectively kill other unwanted scaled fish species during aquaculture [26]. Antimycins are also used as research tools to study the structure and function of cytochromes [27]. More recently antimycins have been shown to be potent and selective inhibitors of the mitochondrial Bcl-2/Bcl-x_L_-related anti-apoptotic proteins [28]. Over-production of Bcl-2/Bcl-x_L_-related proteins in cancer cells confers resistance to multiple chemotherapeutic agents whose mode of action is to trigger apoptosis. A small molecule screen identified antimycins as potent inhibitors of Bcl-2-related proteins where they were shown to bind to the hydrophobic groove [28]. A synthetic derivative of antimycin A_3_, 2-methoxyantimycin A_3_ (Figure 1), no longer inhibits the respiratory chain, but still promotes apoptosis in cells over-producing Bcl-2-related proteins [29]. This suggests antimycin derivatives could be used alongside traditional apoptosis-inducing chemotherapeutics to block drug resistance and kill cancer cells [30]. Therefore, there is significant interest in better understanding the biosynthesis and regulation of antimycins, with a view toward bioengineering improved pharmacological properties for the treatment of drug-resistant cancers in the future.

### The *ant* gene cluster

**Figure 2:**
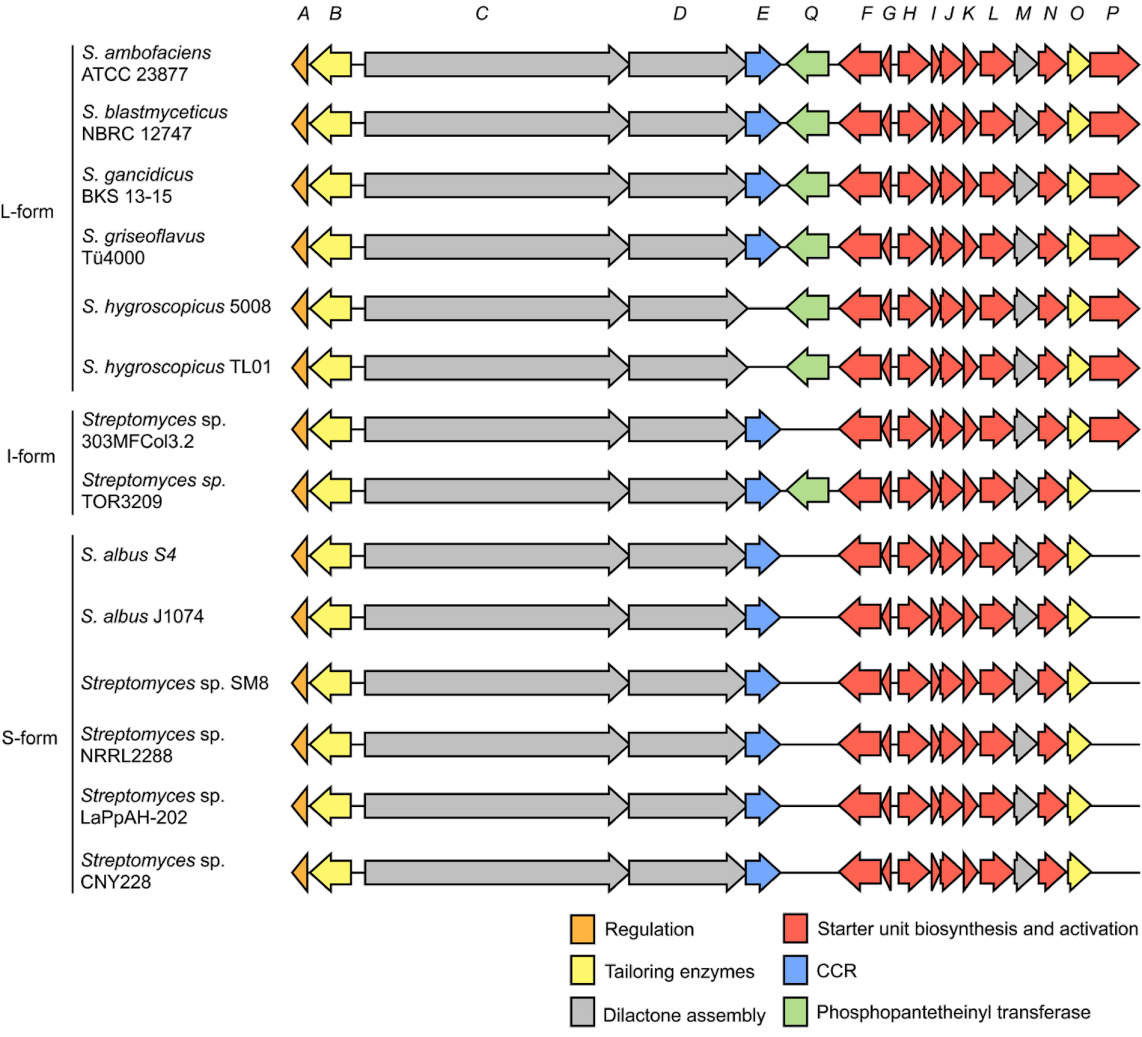
Schematic representation of *ant* biosynthetic gene clusters. L-form *ant* gene clusters are encoded by: *S. ambofaciens* ATCC 23877 (AM238663), *S. blastmyceticus* NBRC 12747 (AB727666), *S. gancidicus* BKS 13-15 (AOHP01000135, AOHP01000134, AOHP01000056, [31]), *S. griseoflavus* Tü4000 (ACFA01000901, ACFA01000902, ACFA01000903, ACFA01000904, ACFA01000905, ACFA01000906, ACFA01000907); *S. hygroscopicus* subsp. *jinggangensis* 5008 (NC_017765); *S. hygroscopicus* subsp. *jinggangensis* TL01 (NC_020895). I-form *ant* gene clusters are encoded by: *Streptomyces* sp. 303MFCol5.2 (ARTR01000061), *Streptomyces* sp. TOR3209 (AGNH01000419, AGNH01000420, AGNH01000421, [32]). S-form *ant* gene clusters are encoded by: *S. albus* S4 (CADY01000091); *S. albus* J1074 (NC_020990); *Streptomyces sp.* SM8 (AMPN01000393, AMPN01000430, AMPN01000050); *Streptomyces* sp. NRRL2288 [25]; *Streptomyces* sp. LaPpAH-202 (ARDM01000016); *Streptomyces* sp. CNY228 (ARIN01000033). The Genbank accession numbers provided correspond to either the complete genome sequence or the contig(s) of draft genome sequences encoding the *ant* gene cluster. It is worth noting that there is a probable sequencing error in the *S. gancidicus* BKS 13–15 cluster which causes the AntD orthologue not to have a stop codon. There is also a probable sequencing error in *S. griseoflavus* Tü4000, which truncates the truncates C1 of AntC into a discrete protein. CCR, crotonyl-CoA reductase.

Since our discovery of the *ant* gene cluster in *S. albus* S4, we and others have identified *ant* gene clusters in 14 out of 117 fully or partially sequenced genomes available in Genbank for the genus *Streptomyces* (Figure 2). The 14 *ant* gene clusters can be classified as long-form (L-form, 17 genes), intermediate-form (I-form, 16 genes) or short-form (S-form, 15 genes). L-form *ant* gene clusters contain two genes, *antP* and *antQ*, which are not encoded by S-form gene clusters [25], and I-form gene clusters contain either *antP* or *antQ*, but not both.

L-form *ant* gene clusters are encoded by six taxa, *S. ambofaciens* ATCC 23877 [7,33], *S. blastmyceticus* NBRC 12747 [25], *S. gancidicus* BKS 13–15 and *S. griseoflavus* Tü4000 [23]. Interestingly, *S. hygroscopicus* subsp. *jinggangensis* 5008 and *S. hygroscopicus* subsp. *jinggangensis* TL01 encode L-form *ant* gene clusters, but do not encode AntE (Figure 2). I-form *ant* gene clusters are encoded by two species, *Streptomyces* sp. 303MFCol5.2 and *Streptomyces* sp. TOR3209, which lack *antQ* and *antP*, respectively (Figure 2). There are six taxa, all highly related to *S. albus* S4, that encode S-form *ant* gene clusters: *S. albus* S4 [7], *S. albus* J1074 [7], *Streptomyces sp.* SM8, *Streptomyces* sp. NRRL2288 [25], *Streptomyces* sp. LaPpAH-202 and *Streptomyces* sp. CNY228.

Based on our analysis of *S. albus* S4, the S-form gene cluster is organised into four transcriptional units; *antAB*, *antCDE*, *antFG* and *antHIJKLMNO* (Seipke and Hutchings, unpublished results; Figure 2). The *antFGHIJKLN* genes encode the biosynthesis pathway for the unusual starter unit, 3-aminosalicylate, *antCD* encode the hybrid NRPS/PKS machinery and *antE* and *antM* encode a crotonyl-CoA reductase and a discrete ketoreductase, respectively. The *antB* and *antO* genes encode tailoring enzymes and *antA* encodes an extracytoplasmic function (ECF) RNA polymerase σ factor named σ^AntA^. The additional genes found in the 17 gene L-form and I-form *ant* gene clusters are *antP* and *antQ*, which encode a kynureninase and phosphopantetheinyl transferase, respectively [25] (Figure 2).

The availability of 14 *ant* gene clusters will facilitate a better understanding of how the different forms of the *ant* gene cluster evolved. More work in this area is required, however it is tempting to speculate that the L-form *ant* gene cluster is the ancestral gene cluster and *antP* and *antQ* were lost, giving rise to the S-form gene cluster. The I-form *ant* gene clusters encoded by *Streptomyces* 303MFCol5.2 and *Streptomyces* sp. TOR3209, which lack *antP* and *antQ,* respectively could be midway points toward evolving into S-form clusters, which presumably use the kynureninase involved in tryptophan catabolism and a phosphopantetheinyl transferase encoded elsewhere in the genome to compensate for the loss of *antP* and *antQ*, respectively.

### Biosynthesis of the antimycin dilactone core

**Figure 3:**
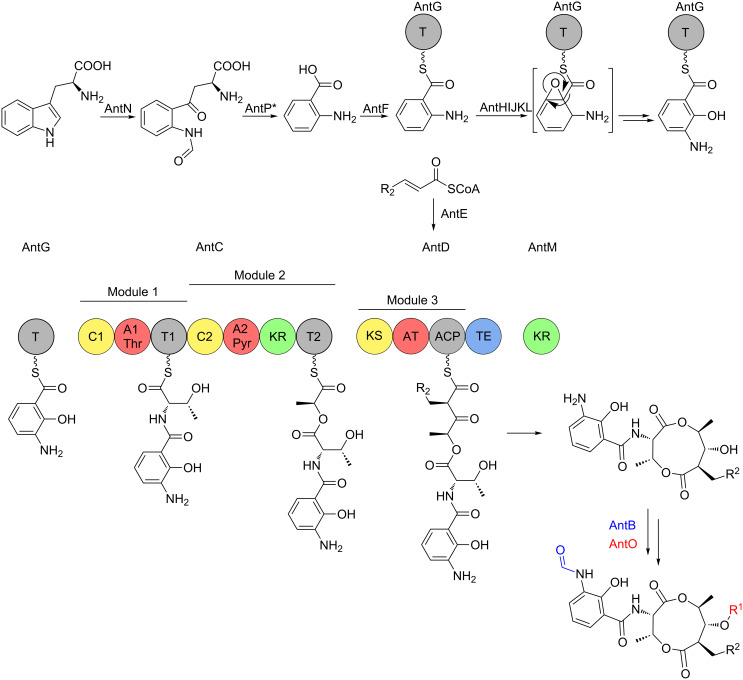
Proposed biosynthetic pathway for antimycins. The antimycin biosynthetic pathway is described in detail in the text. C = condensation; A, adenylation; T, thiolation; KR, ketoreduction; KS, ketosynthase; AT, acyltransferase; ACP, acyl carrier protein; TE, thioesterase; *AntP is a pathway-specific kynureninase that is encoded by L- and I-form *ant* gene clusters.

Antimycins are produced by a hybrid non-ribosomal peptide synthetase (NRPS)/polyketide synthase (PKS) assembly line for which the complete biosynthetic pathway has been proposed [25,34] (Figure 3). The biosynthesis of antimycins involves the activities of fourteen proteins, AntBCDEFGHIJKLMNO. The biosynthesis begins with the opening of the indole ring of tryptophan by a pathway-specific tryptophan-2,3-dioxygenase, AntN, to produce *N*-formyl-L-kynurenine. For L-form *ant* gene clusters, *N*-formyl-L-kynurenine is likely converted to anthranilate by the pathway-specific kynureninase, AntP, whereas S-form gene clusters lack AntP and likely use the kynureninase involved in primary tryptophan metabolism. Anthranilate and not *N-*formylanthranilate is activated by the acyl-CoA ligase protein, AntF and loaded onto its cognate carrier protein, AntG [34]. Once loaded onto AntG, anthranilate is converted to 3-aminosalicylate by a multicomponent oxygenase, AntHIJKL [33–34]. The anthraniloyl-S-AntG carboxylic acid-CoA thioester undergoes a never before seen 1,2-shift. Spiteller and colleagues suggested that AntHIJKL promotes this reaction via an epoxide intermediate similar to a reaction in phenylacetate catabolism [35] resulting in hydryoxylation of C-2 [33]. 3-Aminosalicylate serves as the starter unit and is presented to the NRPS, AntC. The AntC protein possesses two modules organised as follows: C1-A1-T1-C2-A2-KR-T2. The A1 domain activates and loads threonine onto T1, followed by condensation of 3-aminosalicylate and threonine promoted by C1. The A2 domain activates and loads pyruvate onto T2. Pyruvate is subsequently stereospecifically reduced by the KR domain and condensed with threonine by C2. The PKS, AntD posseses one module composed of the domains KS-AT-ACP-TE. The AT domain promotes the transfer of a 2-carboxylated acyl-CoA to ACP. The acyl-CoAs that are utilised by AntD^AT^ are the product of AntE, a crotonyl-CoA reductase homologue, which biosynthesises 'unusual' acylmalonyl-CoAs utilised as PKS extender units [34,36–37]. The AntD AT domain is promiscuous and accepts multiple acylmalonyl-CoAs. In combination with AntE, AntD^AT^ is the source of the large chemical diversity observed at position R^2^ within the antimycin family. Interestingly, *S. hygroscopicus* subsp. *jinggangensis* 5008 and *S. hygroscopicus* subsp. *jinggangensis* TL01 do not encode AntE, suggesting that these strains produce antimycins with less chemical diversity at the R^2^ position (Figure 2 and Figure 3). The KS domain catalyses the decarboxylative condensation between the aminoacyl thioester attached to AntC^T2^ and the 2-carboxy-acyl moiety attached to AntD^ACP^. Next, a discrete ketoreductase, AntM catalyses the stereoselective reduction of the β-keto group, which precedes AntD^TE^ – promoted regiospecific macrolactonisation and release of the nine-membered dilactone. Sandy et al. heterologously produced and purified AntCDEFGM and used building monomers anthranilate, L-threonine, pyruvate, and 2*E*-hexenoyl-CoA to conclusively demonstrate the minimum set of enzymes required for biosynthesis of the antimycin dilactone scaffold in vitro [34].

### Antimycin tailoring genes

All known antimycin gene clusters contain two tailoring enzymes, AntB and AntO (Figure 2). AntB is a discrete acyltransferase, which catalyses a transesterification reaction resulting in the formation of a C-8 acyloxyl moiety and is responsible for generating the chemical diversity at R^1^ (Figure 1). AntB likely performs this reaction after assembly of the dilactone core, as wild-type levels of biosynthetic intermediates possessing a C-8 hydroxyl and not a C-8 acyloxyl were detected in a *∆antB* mutant [38]. In vitro enzymatic synthesis showed that AntB is incredibly promiscuous and is capable of accepting a wide variety of substrates, including an alkyne-containing moiety, which has not been observed previously in the antimycin family [38]. AntB is also able to accept substrates presented by an alternate acyl carrier, *N-*acetylcysteamine (SNAC), though the turnover rate of acyl-SNACs is 100-fold lower than acyl-CoAs [38]. Despite the lower turnover rate, acyl-SNAC substrates are cell-permeable and thus AntB's ability to utilise acyl-SNACs provides the possibility to employ feeding studies to introduce new chemistry at C-8. AntO is a lipase homologue and is predicted to install the *N*-formyl group resulting in the 3-formamidosalicylate moiety. AntO is required for bioactivity against the human pathogenic fungus, *Candida albicans* (Seipke and Hutchings, unpublished results), but the exact time in which AntO installs the *N-*formyl group is unclear and requires investigation.

### Regulation of the *ant* gene cluster

**Figure 4:**
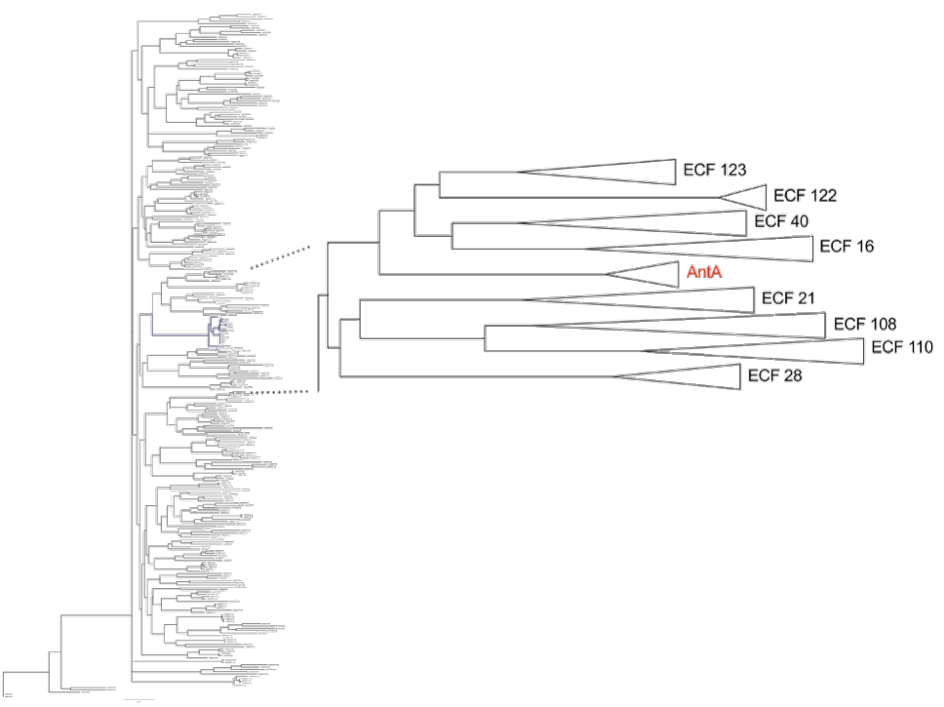
σ^AntA^ comprises a new subfamily of ECF RNA polymerase σ factors. σ^AntA^ amino acid sequences were aligned to amino acid sequences of random representative of each ECF RNA polymerase σ factor subfamily reported by [39] using ClustalΩ [40]. The phylogenetic tree was created using PhyML 3.0 [41] and visualised using FigTree v1.4. The relevant region of the phylogenetic tree is enhanced for visualisation and reveals that all σ^AntA^ protein sequences cluster together and have a distinct phylogenetic lineage and represents a new ECF RNA polymerase σ factor subfamily.

Antimycin biosynthesis is linked to development, as is the case for many *Streptomyces* secondary metabolites. However, antimycin biosynthesis is unusual because all four *ant* operons are highly expressed in substrate mycelium (after 24 hours growth on solid medium) whereas antimycins are not detected until after the production of aerial mycelium when *ant* gene expression is switched off (after 48 hours growth) (Seipke and Hutchings, unpublished results). This lag between gene expression and antimycin production may be due to the complex biosynthetic machinery that needs to be assembled to first make the unusual starter unit 3-aminosalicylate and then finally assemble the antimycin scaffold. The down-regulation of all the *ant* genes in stationary phase also suggests that specific regulatory mechanisms must exist to switch off the expression of all four *ant* operons. Despite this, the 14 *ant* gene clusters contain only a single conserved regulatory gene, *antA*, which encodes an orphan ECF RNA polymerase σ factor named σ^AntA^ (Figure 2). This sigma factor appears to be unique to the 14 known *ant* clusters in the database and they form a new sub-family of ECF sigma factors (Figure 4). Expression of the *antFG* and *antHIJKLMNO* genes is completely dependent on σ^AntA^ and over-expression of *antA* results in *antFGHIJKLMNO* expression in differentiated cultures, suggesting it regulates production of the starter unit, 3-aminosalicylate (Seipke and Hutchings, unpublished results). An unknown regulator, which is not encoded within the *ant* cluster, controls expression of the *antAB* and *antCDE* operons. Despite an earlier report that the S-form *ant* cluster (from *Streptomyces* sp. NRRL 2288) can be heterologously expressed in *S. lividans and S. coelicolor*, we have been unable to replicate this with the cloned S-form *ant* clusters from *S. albus* S4 or *Streptomyces* sp. NRRL 2288 (a kind gift from Professor Wen Liu). This suggests that the regulator of *antAB* and *antCDE* is a transcriptional activator that is encoded outside of the *ant* gene cluster and is absent from the heterologous host strains (Seipke and Hutchings, unpublished results).

Regulation of antibiotic gene clusters by ECF σ factors has only been reported in two rare actinomycete strains and in both cases there is a co-encoded anti-σ factor, which regulates σ factor activity, along with a pathway specific transcriptional activator. In *Microbispora corallina* production of the lantibiotic microbisporicin is regulated by the pathway-specific transcription factor MibR and the ECF σ^MibX^, whose activity is modulated by its cognate anti-σ factor, MibW [8]. Biosynthesis of the lantibiotic planosporicin by *Planomonospora alba* is also regulated by a σ and anti-σ factor pair (PspX and PspW) whose closest homologues are MibX and MibW, respectively, and by the pathway-specific LuxR-family regulator, PspR [42]. There are no anti-σ factors encoded in any of the 14 *ant* gene clusters and no transcriptional regulators other than σ^AntA^ (Figure 2). Production of σ^AntA^ is regulated at the transcriptional level, by an unknown transcription factor, and it appears that σ^AntA^ is rapidly cleared from the cell once transcription of *antA* is switched off (Seipke and Hutchings, unpublished results). One striking feature of the 14 known σ^AntA^ proteins is that they all terminate in Ala–Ala, a well-known signal for the ClpXP protease. Attempts to N-terminally 6xHis-tag σ^AntA^ so that we could detect it using monoclonal anti-His antibodies were unsuccessful because His_6_-σ^AntA^ does not complement the *antA* mutant strain of *S. albus* S4, suggesting the protein is inactive. Furthermore, polyclonal antibodies raised against purified σ^AntA^ reacted with the purified σ^AntA^ protein in immunoblotting experiments, but could not detect σ^AntA^ in whole cell extracts, even from strains over-expressing *antA*, perhaps suggesting the protein is very unstable. Intriguingly, a variant of the *S. albus* S4 σ^AntA^ protein in which the C-terminal Ala–Ala was changed to Asp–Asp resulted in higher levels of expression for the *antFG* and *antHIJKLMNO* operons suggesting this may increase the stability or activity of σ^AntA^ (Seipke and Hutchings, unpublished results). A role for ClpXP in regulating σ^AntA^ activity remains to be proven however.

### Future perspectives: toward bioengineering antimycins with improved pharmacological properties

There is significant interest in bioengineering new antimycin analogues with improved pharmacological properties for use as antifungal therapeutics and to be used to treat cancers, alongside traditional chemotherapy agents. Substantial progress has been made in a relatively short time and has mostly been driven by studies rooted in better understanding the biosynthesis of these unusual molecules.

Initial success toward bioengineering new antimycins was achieved by feeding modified monomer building blocks to batch bacterial fermentations. Spiteller and colleagues showed that by feeding fluoroanthranilates to *S. ambofaciens* ATCC 23877 and *S. odorifer* DSM 40347 cultures they could produce 5-fluoroantimycins and 4-fluoroantimycins [33] (Figure 1). Similarly, feeding 6-fluoro-L-tryptophan to cultures of *Streptomyces* sp. NRRL 2288 resulted in the production of 5-fluoroantimycins [25]. Liu and coworkers analysed the bioactivity of 5-fluoroantimycins and reported they retained potent antifungal activity against *Candida albicans*, but were significantly reduced in cytotoxity in a leukemia P388 mouse cell line compared to the parent compounds [25]. Sandy et al. recently showed AntB can accept both acyl-CoAs and acyl-SNACs to form the C-8 acyloxy group and generate diversity at R^1^ in vitro [38]. Acyl-SNACs are cell permeable, and the ability of AntB to utilise these substrates provides the opportunity to perform feeding studies using synthesised acyl-SNACs to introduce new chemistry at R^1^ [38].

The gene clusters for JBIR-06 (12-membered ring), neoantimycin (15-membered ring), and 18-membered ringed respirantin were recently identified [43–44]. JBIR-06 and neoantimycin inhibit GRB78 chaperone involved in the unfolded protein response [45–46]. Although the DNA sequence for the gene clusters for these ring-expanded antimycins has not yet been made publically available, they all encode the machinery necessary to assemble the 9-membered antimycin core suggesting a common evolutionary past [44]. The vast chemical diversity in the antimycin family together with the recent characterisations of the promiscuous biosynthetic machinery suggest it is possible to use synthetic biology to bioengineer non-natural analogues in large enough quantity to test their efficacies in the clinic. In line with that view, generating a chemistry-dereplicated culture collection of antimycin-type depsipeptide producers to build a library of swappable biosynthetic gene cassettes to introduce new chemical diversity should result in rapid generation of new analogues in the antimycin family.
